# The association between HIV pretreatment drug resistance and virological outcomes in children and adults in sub-Saharan Africa: A systematic review and meta-analysis

**DOI:** 10.1371/journal.pone.0300456

**Published:** 2024-04-16

**Authors:** Ebako Ndip Takem, Christopher Coox, Judith Shang, Clement Ndongmo, Emily Kainne Dokubo

**Affiliations:** Centers for Disease Control and Prevention (CDC), Cameroon; Nigerian Institute of Medical Research, NIGERIA

## Abstract

**Introduction:**

Pretreatment drug resistance (PDR) could occur in antiretroviral treatment (ART) naïve individuals, those previously exposed to ART, or individuals re-initiating ARV after a long period of interruption. Few studies have shown its association with virological outcomes, although inconsistent. The objective of this review was to provide a synthesis of the association between PDR and virological outcomes (virological failure or suppression).

**Methods:**

This report is presented following the Preferred Reporting Items for Systematic Reviews and Meta-Analyses (PRISMA) guidelines. The method was subdivided into three main phases: record identification, screening, and report inclusion. Record identification consisted of an initial search with search term “HIV pretreatment drug resistance”. Another search was done using terms “Pretreatment drug resistance OR pre-treatment drug resistance OR Pretreatment drug resist* OR pre-treatment drug resist* OR pretreatment antiretroviral resistance OR pretreatment medic* OR pretreatment medic* resist*” and a list of all the countries in sub-Saharan Africa. After the electronic search, studies were screened from full list based on their title and abstract and then full articles retrieved and studies were assessed based on set criteria. Inclusion criteria involved observational studies that report the association between PDR and virological failure. Data from trials that reported the association were also included. Published articles like modelling studies and reviews, and studies with data that had been previously included in the review were excluded. The Mantel Haenszel method with odds ratios was used for synthesis (meta-analyses) with the weights of each study which depends on the number of events and totals.

**Results:**

A total of 733 records(studies) were obtained from all database search of which 74 reported on PDR, virological outcomes in sub-Saharan Africa (SSA). Out of the 74 articles, 11 were excluded and 26 did not explicitly report data needed, and 5 did not meet the inclusion criteria. Of the remaining 32 studies, 19 studies that had complete data on the number of participants with PDR and no PDR according to virological failure (VF) were included in the metanalyses. The pooled results from eleven (13) of these studies showed those with PDR had higher odds of virological failure compared to those without PDR OR 3.64[95% CI 2.93, 4.52]. The result was similar when stratified in adults and in children. In six (6) studies that had Virological suppression (VS) as outcome, there was a reduction in the odds of VS in those with PDR compared to those without PDR, OR 0.42 (95% CI 0.30, 0.58).

**Conclusion:**

In conclusion, this systematic review indicates that PDR increases the risk of virological failure in sub-Saharan Africa. The risk could be reduced by PDR monitoring for NNRTIs and INSTIs.

## Introduction

HIV drug resistance occurs when there is a change in the virus genome which makes it less responsive to antiretroviral (ARV) treatment. This resistance could develop in those who are currently receiving ARVs (acquired drug resistance-ADR). On the other hand, pretreatment drug resistance (PDR) could occur either in ART naïve persons, those previously exposed to ART (Pre-exposure prophylaxis -PrEP, Prevention of Mother to Child Transmission-PMTCT) or individuals re-initiating ARV after a long period of interruption and without documented virological failure-VF [1]. In ART naïve persons, PDR occurs as a result of transmission of resistant strains, and this is termed transmitted drug resistance (TDR). Pretreatment drug resistance can occur to commonly used ARV classes, which include Nucleoside reverse transcriptase inhibitor (NRTI), Non-nucleoside reverse transcriptase inhibitor (NNRTI), protease inhibitor (PI), and integrase inhibitor strand transfer inhibitor (INSTI) [2].

The prevalence of PDR in adults, obtained from nationally representative surveys conducted in 4 countries in SSA (Cameroon, Namibia, Uganda, and Zimbabwe) from 2015–2016, indicates 3 of the 4 countries had rates above 10% [1]. This was mostly driven by NNRTI PDR with prevalence ranging from 8.1% in Cameroon to 15.4% in Uganda [1]. Disease modelling predicts that as more individuals are on ARV, there will be less HIV transmission, and increase in prevalence of HIVDR, all due to treatment failure and transmitted resistance [3, 4]. Thus, the prevalence of PDR is likely to increase with more individuals on ARV treatment. The prevalence of PDR appears to be higher in women compared to men, and in those with previous exposure to ARV compared to the ARV naïve [1].

Different definitions of virological outcomes have been used to evaluate the effect of PDR, which include viral load suppression (VLS), VF, treatment failure (TF), time to VLS or VF. Previous studies in SSA have shown an association between PDR and VF [5–16], VLS [13, 17–20],time to VF [11], and time to VLS [21, 22]. Concerning the association between ARV class-specific PDR and VF, the association has been mostly observed with NRTIs and NNRTIs and less with PIs [1, 6]. This is not unexpected, as these are the drug classes that had been used for a longer duration of time by majority of ARV individuals. However, this should be interpreted with caution as some reports have shown an interaction between ARV class types [23]. In addition, resistance to one ARV class could lead to resistance to another class. Some examples of these interactions include NNRTI resistance has been shown to lead to PI failure [24] but may not necessarily lead to VF if efavirenz (EFV) is present [6]. PDR was associated with VF in patients prescribed partially active ARVs (NNRTI) to which they have mutations and was not associated with VF in patients receiving fully active ARVs for which they had no resistant mutations [7]. Participants with NNRTI+NRTI resistance who received non tenofovir (TDF) regimens had increased risk of VF, while participants with NNRTI-NRTI regimens who received TDF regimens did not have increased risk. Those on regimens of NNRTI only (no NRTI) with TDF had increased risk of VF [9]. NNRTI resistance was associated with reduction in efficacy of dolutegravir (DTG) regimen [25]. Transmitted drug resistance (TDR) to NNRTI and lamivudine (3TC) was associated with VF in Kenyan ARV naïve adults [5]. In children, low level mutations to NNRTI did not affect the use of nevirapine (NVP) [26].

The relative contributions of potentially modifiable risk factors to viremia (PDR, non-adherence and low-level viremia) have also been evaluated in SSA [10]. PDR against NNRTI-class ARVs and non-adherence are mainly associated with virological non-suppression. Virological rebound episodes are associated with low-level viremia (LLV) and non- adherence [10]. Overall, non-adherence accounted for a larger portion of viral non suppression [10]. In addition to VF, PDR has been shown to be associated with the development of acquired drug resistance [12, 18]. Although many studies have elucidated the association between PDR and virological failure, there is some indication that not all mutations lead to VF [13]. Furthermore, the effect of resistance on VF it is not known when PIs are used with other drug classes as baseline [25].

It is important to quantify the association between PDR and viral load outcomes and estimate the contribution of PDR to viral failure in different settings with variable levels of PDR. Examining PDR could also help identify drug classes for which the virus is presenting resistance, which could inform decisions about switching to newer regimen. Quantifying PDR gives an idea of the effectiveness of the currently used ARVs locally.

This systematic review would provide pooled data for SSA, which would give an idea of the magnitude of the public health problem. It will provide a synthesis of the association between PDR and viral load outcomes (Virological failure or suppression) which will help estimate the impact of PDR to VF which will help improve the response to ARV and hence reduce morbidity and mortality. In addition, this will provide more information about the specific effect of ARV class-specific mutations on VF for which data from previous studies is limited. There will be synthesis of association between PDR and VL outcomes in children which is sparse in available literature.

This review report is presented following the Preferred Reporting Items for Systematic Reviews and Meta-Analyses (PRISMA) guidelines [27].

## Methods

### Design

This systematic review assessed the available literature on the topic of HIV pretreatment drug resistance (PDR) and virological outcomes in sub-Saharan African countries. The methods could be grouped into 3 main chronological phases which include record identification, screening, and report inclusion. These phases were conducted independently by two of the manuscript authors.

### Record identification

Record identification consisted of conducting a literature search using the PubMed electronic database in April 2021.

An initial search was done using the search term “HIV pretreatment drug resistance” to include a wide range of articles without restriction.

Another search was conducted using the following search terms:

Pretreatment drug resistance OR pre-treatment drug resistance OR Pretreatment drug resist* OR pre-treatment drug resist* OR pretreatment antiretroviral resistance OR pretreatment medic* OR pretreatment medic* resist*.

The search was conducted in April 2021 without restrictions on date of publication.

HIV OR AIDS.

Viral suppression OR virological suppression OR virological failure OR viral un-suppression OR viral unsuppress* OR treatment failure OR failure.

Angola OR Benin OR Botswana OR Burkina Faso OR Burundi OR Cabo Verde OR Cameroon OR Chad OR Cote d’Ivoire OR Congo OR Central African Republic OR Comoros OR Democratic Republic of Congo OR Djibouti OR ESwatini OR Ethiopia OR Eritrea OR Equatorial Guinea OR Guinea Bissau OR Gabon OR Gambia OR The Gambia OR Ghana OR Guinea OR Kenya OR Lesotho OR Liberia OR Madagascar OR Malawi OR Mauritania OR Mali Mozambique OR Nigeria OR Niger OR Namibia OR Rwanda OR Sao Tome OR Senegal OR Seychelles OR Mauritius OR Sierra Leone OR South Africa OR Swaziland OR Somalia OR Sudan OR South Sudan OR Tanzania OR Togo OR Uganda OR Zambia OR Zimbabwe.

An additional new search was conducted in January 2023 using the following 5 databases which include 1) ACADEMIC SEARCH COMPLETE, 2) EMBASE, 3) LILACS, 4) MEDLINE, 5) PROQUEST.

In addition, a PubMed search was repeated in 2023 to see if additional articles were obtained.

Search results were imported to Review Manager package (Review Manager (RevMan) [Computer program]. Version 5.4. The Cochrane Collaboration, 2020.) for the management of reports, references and analyses including metanalyses.

### Screening

After the electronic search, the records were screened. In the first stage, studies were screened from the search list based on their title and abstract. In the second stage, full articles were retrieved, and studies were assessed and included following set criteria.

### Report inclusion

#### Inclusion criteria

- *Type of studies*. Study designs included were cohort studies, case control studies, cross-sectional studies, and ecological studies. Studies from trials that also reported the relationship between PDR and virological failure were also retrieved.
- *Population*. Only papers that reported pretreatment drug resistance and virological outcomes in sub-Saharan African countries were included. PDR includes TDR transmitted at time of infection or through PMTCT/ PrEP. This includes, but is not limited to, studies involving data collection on PDR in health facilities in children, adults, or both.
- *Intervention*. The exposure is those with PDR. PDR could be resistance to any class of ARV (NNRTI, NNRTI PI), resistance to a single ARV class, or both.
- *Comparators*. PDR was accessed according to virological failure and virological suppression in children and in adults.
- *Types of outcomes*. Virological failure was defined as VL≥ 1000 copies and virological suppression defined as VL <1000 copies at 6 months, or 12 months, 18 months after ART initiation. Other cut-offs for virological failure/suppression were used in the different studies. Data from the studies was gathered and used for analysis and write-up.

#### Exclusion criteria

Studies with one of the following were excluded: Data (the same period) has been used previously in another publication already selected in the review, Publication type is modelling or systematic review, or case reports.

### Data analyses

Once all relevant papers that matched the inclusion criteria were identified, data on the number of participants with PDR, without PDR, virological outcomes in those with PDR and those without PDR, were extracted from the full articles and entered into the RevMan package (Review Manager (RevMan) [Computer program]. Version 5.4. The Cochrane Collaboration, 2020). This was done independently by two of the study investigators and results verified and corrected in case of discrepancies. Odds ratios were used to examine the association between PDR and virological outcomes for each study. The Mantel Haenszel method was used for the synthesis to get the overall odds ratios (metanalyses). The weights for each study were estimated considering the number of events and the totals. Briefly, each study was taken as separate strata and then the estimates were calculated and a test for heterogeneity was used to evaluate the heterogeneity between the strata. The individual study odds ratios and their confidence intervals and the pooled estimates were used to develop Forest plots. The data for the numbers with PDR and the total numbers was used to estimate the prevalence of individual studies and the pooled prevalence (metanalyses) using STATA version 18 (College Station, Texas).

The risk of bias in each study was evaluated using a tool with 10 items as described by Hoy et al. [28].

## Results

### Study selection

**Fig 1:**
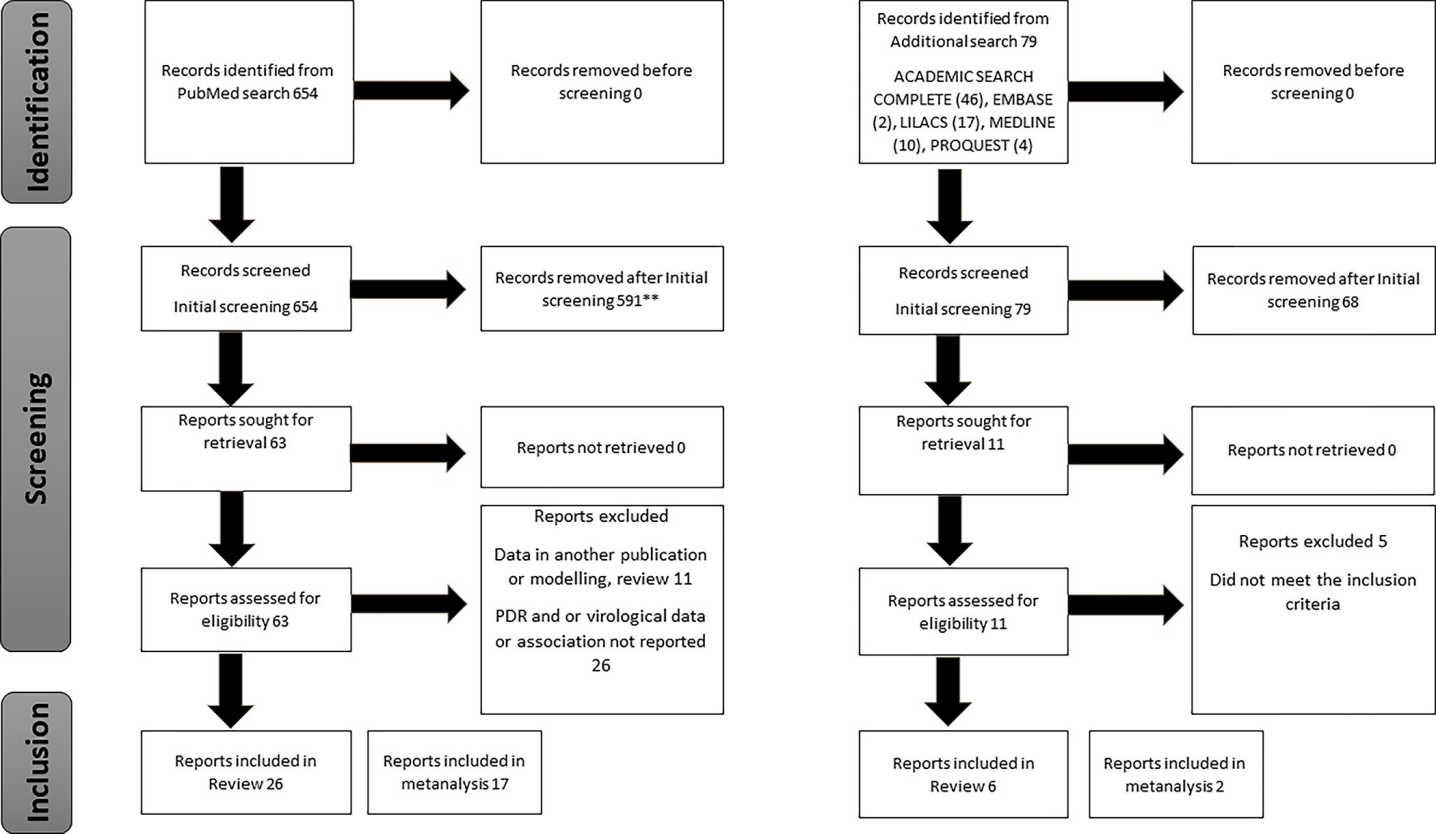
Flow diagram*. *Figure presented following PRISMA guidelines **17 out of 599 articles were retrieved after getting them from references in the published articles and 3 were included.

A total of 654 records (articles) were obtained from the PubMed electronic database search. No duplicates were found, and no records were removed prior to screening. The initial screening involved looking at the records list to see if they were focused on pretreatment drug resistance and virological outcome, as well as being conducted in a sub-Saharan African country. A total of 63 studies met these criteria and were retained for full report/article retrieval, from published literature. No grey literature record was included. These full articles were thoroughly read to determine if they met the eligibility or inclusion criteria. Out of the 63 studies, 11 were excluded for one of the following reasons: 1) the data had been reported in other studies included in this review, 2) the publication type was not original research i.e., review or modelling. Twenty-six (26) out of the 63 studies did not explicitly report PDR data, virological data, or their association. The remaining 26 of the 63 studies were included in this systematic review. Out of these, 17 studies reported complete data on number of participants with PDR and no PDR, and virological outcomes in each category (Fig 1).

The additional search involving five databases retrieved 79 articles- Academic search complete (46), EMBASE (2), LILACS (17), MEDLINE (10), PROQUEST (4). Initial screening to look for records focused on pretreatment drug resistance and virological outcome in SSA. A total of eleven (11) articles were finally retained while leaving out articles already retrieved from PubMed search. The 11 full articles were obtained and detailly read to verify the inclusion criteria. Out of these, five (5) were excluded for one of the following reasons 1) the article looked at PDR rates with or without virological outcome but did not explicitly present their association, 2) the study location in SSA was not explicitly mentioned. Finally, a total of six (6) articles were included in the systematic review from which two were included in metanalysis (Fig 1).

Totally from the two searches, 733 (654+79) records were obtained of which 74 (63+11) reported on PDR, virological outcomes in sub-Saharan Africa (SSA). Out of the 74 articles, 11 were excluded, 26 did not explicitly report data needed, and 5 did not meet the inclusion criteria. Of the remaining 32 (26+6) studies, 19 (17+2) studies that had complete data on the number of participants with PDR and no PDR according to virological failure (VF) were included in the metanalyses.

### Characteristics of included studies

A total of 32 studies [5–16, 18–21, 23–26, 29–40] that looked at the association between PDR and virological outcome (VF/TF, VLS) were included in this review (Table 1).

**Table 1:** Characteristics of included studies for systematic review.

study	Country	Year	Age	N	Study design	Study population	Resistance Mutation Classes
Boerma 2016 [29]	Nigeria	2012–2013	≤ 12 years	90	Cohort	HIV infected children	NRTI+NNRTI
Chimukangara 2020 [24]	South Africa	SAPiT 2005–2008 2009–2013 (TRuTH)	≥18 years	170	Nested case control (from SAPiT trial and TRuTH study)	HIV/TB co-infected	NRTI, NNRTI
Chung 2014 [5]	Kenya	2006	32–43 IQR	386	Cohort from trial	HIV infected	NNRTI
Chung 2020a [6]	Kenya	2013–2014	2-≥50 years	987	Cohort from trial	≥2 years HIV infected	NNRTI
Crowell 2017 [23]	Mali	2010–2013	<10 years	150	Cohort	HIV infected children	NRTI+NNRTI
Hamers 2012 [7]	Kenya, Nigeria, South Africa, Uganda, Zambia and Zimbabwe	2007–2009	≥18 years	2579	Cohort (multicentre)	HIV infected	NRTI+NNRTI
Hassan 2019 [8]	Kenya	2008–2013	28.9–42 years IQR	48	Cohort	HIV infected	NRTI+NNRTI
Hong 2015 [30]	Namibia	2009	>18 years	394	Cohort	HIV infected	NNRTI, PI
Hunt 2011 [26]	South Africa	2005–2007	<24 months	255	Cohort	HIV infected children	NRTI, NNRTI, PI
Inzaule 2019a [9] (similar data from Boender 2015 and Hamers 2012) but looked at NNRTI specific PDR on VF				1941			
Inzaule 2020 [10]	Kenya, Nigeria, South Africa, Uganda, Zambia and Zimbabwe	2007–2014	≥ 18 years	1935	Cohort	HIV infected	continuation with data from Hamers 2012
Kantor 2015 [11]	Brazil, Haiti, India, Malawi, Peru, South Africa, Thailand, US and Zimbabwe	2005–2007	>18 years	466	Nested case cohort	HIV infected	NRTI, NNRTI and PI based combination
Kityo 2017 [12]	Uganda	2010–2011	≤12 years	317	Cohort (multicentre)	HIV infected	NRTI+NNRTI, PI based
Lee 2014 [13]	Uganda	2002–2004 (AMU), 2005–2010 (MBA)	30–40 years IQR	497	Cohort (UARTO)	HIV infected	NRTI, NNRTI
McCluskey 2018 [21]	Uganda	2013–2015	≥ 18 years	738	Cohort (UARTO)	HIV infected	NRTI, NNRTI
Milne 2019 [14]	Kenya	N/M*	≥18 years	169 (OLA)	Cohort	HIV infected women	NRTI NNRTI. Not mentioned study period but indicated in Beck 2020
Moorthy 2011 [31]	South Africa		2–24 months	124	Cohort from trial data (NEVEREST)	HIV infected children	NRTI, NNRTI
Mungati 2016 [32]	Zimbabwe	2008–2010	30–44 IQR	1398	Cross-sectional analyses data taken from cohort	HIV infected	NRTI, NNRTI
Mzingwane 2016 [33]	South Africa	2013–2014	23–69	78	Cohort	HIV infected	NRTI, NNRTI
Reynolds 2012 [34]	Uganda	2004–2009	14–32 years (IQR)	41	Cohort	HIV infected	NRTI, NNRTI
Siedner 2020 [25]	South Africa	N/M	>12 years	852	Cohort from trial data (ADVANCE)	HIV infected non pregnant	NRT+NNRTI, DTG based
Soeria-Atmadja 2020 [18]	Uganda	2015–2016	3–12 years	85	Cohort (GENEFA)	HIV infected children	NRTI+NNRTI
Tadesse 2019 [20]	Ethiopia	N/M	3–12 years	51	Cohort	HIV infected Children	NNRTI
Taffa 2018 [19]	Namibia	2015 and 2016	≥18 years	384	Cross-sectional survey but follow VL done which means cohort	HIV infected	NRTI, NNRTI, PI
Telele 2018 [35]	Ethiopia	2009–2011	≥14 years	461 (SDRMs)	Cohort	HIV infected	NRTI+NNRTI, PI
Towler 2010 [36]	Uganda	2004–2006	24–148 months	74	Cohort	HIV infected Children	NRTI+NNRTI
Hermans 2022 [15]	South Africa	2015–2017	>18 years	154	Cohort (data from trial)	HIV infected adults	NRTI+NNRTI
Beesham 2022 [37]	South Africa	2015–2018	16–35 years	275	Cohort (data from trial)	HIV infected women (negative before)	NRTI+NNRTI
Crowell 2021 [38]	Uganda, Kenya, Tanzania, Nigeria	2013–2019	> = 18 years	801	Cohort	HIV infected adults, ART naïve, viremic (VL>1000)	NRTI, NNRTI, PI
Kuhn 2014 [39]	South Africa	2011	< 2 years	230	Cohort	HIV infected infants	NRTI, NNRTI
Moraka 2021 [40]	Botswana	N/M	1–32 days	27	Cohort	HIV infected infants	NRTI, NNRTI, PI, INSTI
Lippman 2020 [16]	South Africa	2014	18–49 years	107	Cross-sectional survey	HIV infected adults	NRTI, NNRTI, PI

N/M = Not Mentioned

*Study period not mentioned in paper but 2010 mentioned in Beck 2020

A total of 19 studies that explicitly presented data on all the following were eligible for meta-analyses: number of participants with PDR, number of participants without PDR, and virological outcomes in those with PDR and those without. This corresponded to a total of 8848 participants (Figs 3 and 4). There were 13 studies on virological failure as outcome, with 7354 participants and 2356 events. In addition, there were 6 studies with viral load suppression as outcome, with 1494 participants and 1016 events.

Out of the 13 studies with VF as outcome, one study contributed to more than one quarter of the study participants (weight 26.8%); a multicenter study in 6 African countries. The study that contributed to the highest proportion of events was 62% (1288/2073) which was a study conducted in HIV infected adults in Zimbabwe (Fig 3).

Out of the 6 studies with VLS as outcome, one study contributed to the highest number of participants corresponding to a weight of 55.7% and the highest number of events (Fig 4).

A total of 6 out of the 19 studies included data on children <18 years. Thirteen studies involved adults of which two of the studies had a combination of adults and children (Figs 3 and 4).

The study period ranged from 2004–2019 with majority of the studies conducted in East and Southern Africa. One study was from Mali and data from Nigeria was included only as part of a multicenter study. In terms of study design, most of the studies were cohort studies (including those from trials) and fewer were nested case control studies.

Most of the patients were seen at baseline when samples were collected for resistance tests and then followed up at different time points after ARV initiation. The different time points included 14 weeks, 6 months(m), 12m, 24m, 18 m for VF, and 24 weeks, 48w, 12m, 24m, 36m, 6-12m, 180 weeks, all after treatment initiation.

The ARVs prescribed included mostly combinations comprising NRTI and NNRTI and few with PI based combinations (Table 1).

### Risk of bias in studies

In cohort studies that were included, the percentage loss to follow-up was generally low (Table 2), indicating a minimal risk of bias [41, 42].

**Table 2:** Risk of bias of studies included.

study	Country	Age	N	Study design	Study population	Risk of bias	Bias Assessment tool
Boerma 2016 [29]	Nigeria	≤ 12 years	90	Cohort	HIV infected children	Minimal risk of bias 10% loss to follow up (LTFU)-low	Low
Chimukangara 2020 [24]	South Africa	≥18 years	170	Nested case control (from SAPiT trial and TRuTH study)	HIV/TB co-infected	Minimal risk of bias Cases and controls comparable Unclear whether cases and controls (nested) representative of the source population	Low
Chung 2014 [5]	Kenya	32–43 IQR	386	Cohort from trial	HIV infected	Borderline minimal risk of bias 20% LTFU (borderline)	Low
Chung 2020a [6]	Kenya	2-≥50 years	987	Cohort from trial	≥2 years HIV infected	19% LTFU	Low
Crowell 2017 [23]	Mali	<10 years	150	Cohort	HIV infected children	20% LTFU	Low
Hamers 2012 [7]	Kenya, Nigeria, South Africa, Uganda, Zambia and Zimbabwe	≥18 years	2579	Cohort (multicentre)	HIV infected	18% LTFU at 12 months	Low
Hassan 2019 [8]	Kenya	28.9–42 years IQR	48	Historical Cohort	HIV infected	Those with at least 3 samples enrolled. LTFU not applicable.	Low
Hong 2015 [30]	Namibia	>18 years	394	Cohort	HIV infected	78% follow up (i.e., LTFU 22%)	Low
Hunt 2011 [26]	South Africa	<24 months	255	Cohort	HIV infected children	Follow up not mentioned	Low
Inzaule 2019a [9] (similar data from Boender 2015 and Hamers 2012) but looked at NNRTI specific PDR on VF			1941			LTFU not mentioned	
Inzaule 2020 [10]	Kenya, Nigeria, South Africa, Uganda, Zambia and Zimbabwe	≥ 18 years	1935	Cohort	HIV infected	LTFU not mentioned	Low
Kantor 2015 [11]	Brazil, Haiti, India, Malawi, Peru, South Africa, Thailand, US and Zimbabwe	>18 years	466	Nested case cohort	HIV infected	LTFU not mentioned	Low
Kityo 2017 [12]	Uganda	≤12 years	317	Cohort (multicentre)	HIV infected children	18% LTFU	Low
Lee 2014 [13]	Uganda	30–40 years IQR	497	Cohort (UARTO)	HIV infected	Not mentioned	Low
McCluskey 2018 [21]	Uganda	≥ 18 years	738	Cohort (UARTO)	HIV infected	Differential LTFU between groups PDR and non PDR	Low
Milne 2019 [14]	Kenya	≥18 yrs	169 (OLA)	Cohort	HIV infected women	Low LTFU (<5%)	Low
Moorthy 2011 [31]	South Africa	2–24 months	124	Cohort from trial data (NEVEREST)	HIV infected children	LTFU rate not mentioned	Low
Mungati 2016 [32]	Zimbabwe	30–44 IQR	1398	Cross-sectional analyses data taken from cohort	HIV infected	Not explicitly mentioned	Low
Mzingwane 2016 [33]	South Africa	23–69	78	Cohort	HIV infected	20% LTFU	Low
Reynolds 2012 [34]	Uganda	14–32 years (IQR)	41	Cohort	HIV infected	NRTI NNRTI	Low
Siedner 2020 [25]	South Africa	>12 years	852	Cohort from trial data (ADVANCE)	HIV infected non pregnant	LTFU appears low	Low
Soeria-Atmadja 2020 [18]	Uganda	3–12 years	85	Cohort (GENEFA)	HIV infected children	<10% LTFU	Low
Tadesse 2019 [20]	Ethiopia	3–12 years	51	Cohort	HIV infected Children	LTFU not mentioned	Low
Taffa 2018 [19]	Namibia	≥18 years	384	Cross-sectional survey but follow VL done which means cohort	HIV infected	Not mentioned	Low
Telele 2018 [35]	Ethiopia	≥14 years	461 (SDRMs)	Cohort	HIV infected	LTFU <20%	Low
Towler 2010 [36]	Uganda	24–148 months	74	Cohort	HIV infected Children	Not mentioned	Low
Hermans 2022 [15]	South Africa	>18 years	154	Cohort (data from trial)	HIV infected adults	LTFU	Low
Beesham 2022 [37]	South Africa	16–35 years	275	Cohort (data from trial)	HIV infected women (negative before)	Not mentioned	Low
Crowell 2021 [38]	Uganda, Kenya, Tanzania, Nigeria	> = 18 years	801	Cohort	HIV infected adults, ART naïve, viremic (VL>1000)	Not mentioned	Low
Kuhn 2014 [39]	South Africa	< 2 years	230	Cohort	HIV infected infants	Not mentioned	Low
Moraka 2021 [40]	Botswana	1–32 days	27	Cohort	HIV infected infants	Not mentioned	Low
Lippman 2020 [16]	South Africa	18–49 years	107	Cross-sectional survey	HIV infected adults	70% samples sequenced	Low

In the case-control study, the risk of bias was low- those with virological outcomes were less likely to be different from the general population from which they came.

The assessment tool indicated that the studies had low risk of bias (Table 2).

## Results of individual studies

Twelve (12) out of thirteen (13) studies showed an increase in the odds of VF in those with PDR compared to those without PDR, and one study showed a decrease (Fig 3). There was evidence of some variability between the individual studies (test for heterogeneity Chi2 50.07, p<0.00001). However, looking at the pediatric studies only, there was less variability (Heterogeneity test Ch2 2.94, p = 0.23).

### Results of metanalyses

#### Prevalence of PDR

**Fig 2:**
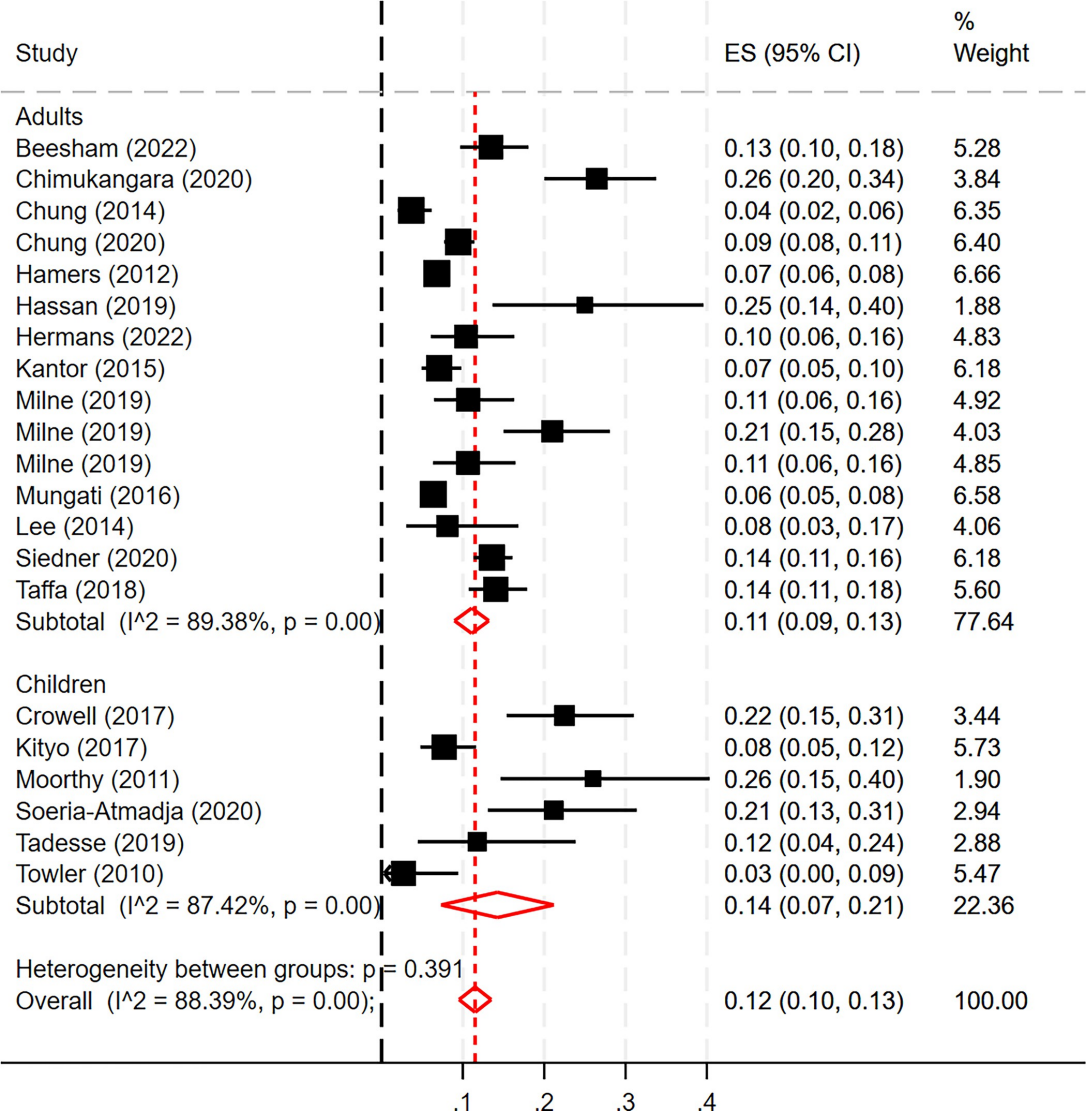
Prevalence of PDR (metanalysis).

The prevalence of PDR ranged from 4–26% in adults with a pooled prevalence of 11% [0.11(95% CI 0.09–0.13)], and a prevalence of 3–26% in children with a pooled prevalence of 14% [0.14(95% CI 0.07–0.21)] (Fig 2).

#### Association between PDR and VF

**Fig 3:**
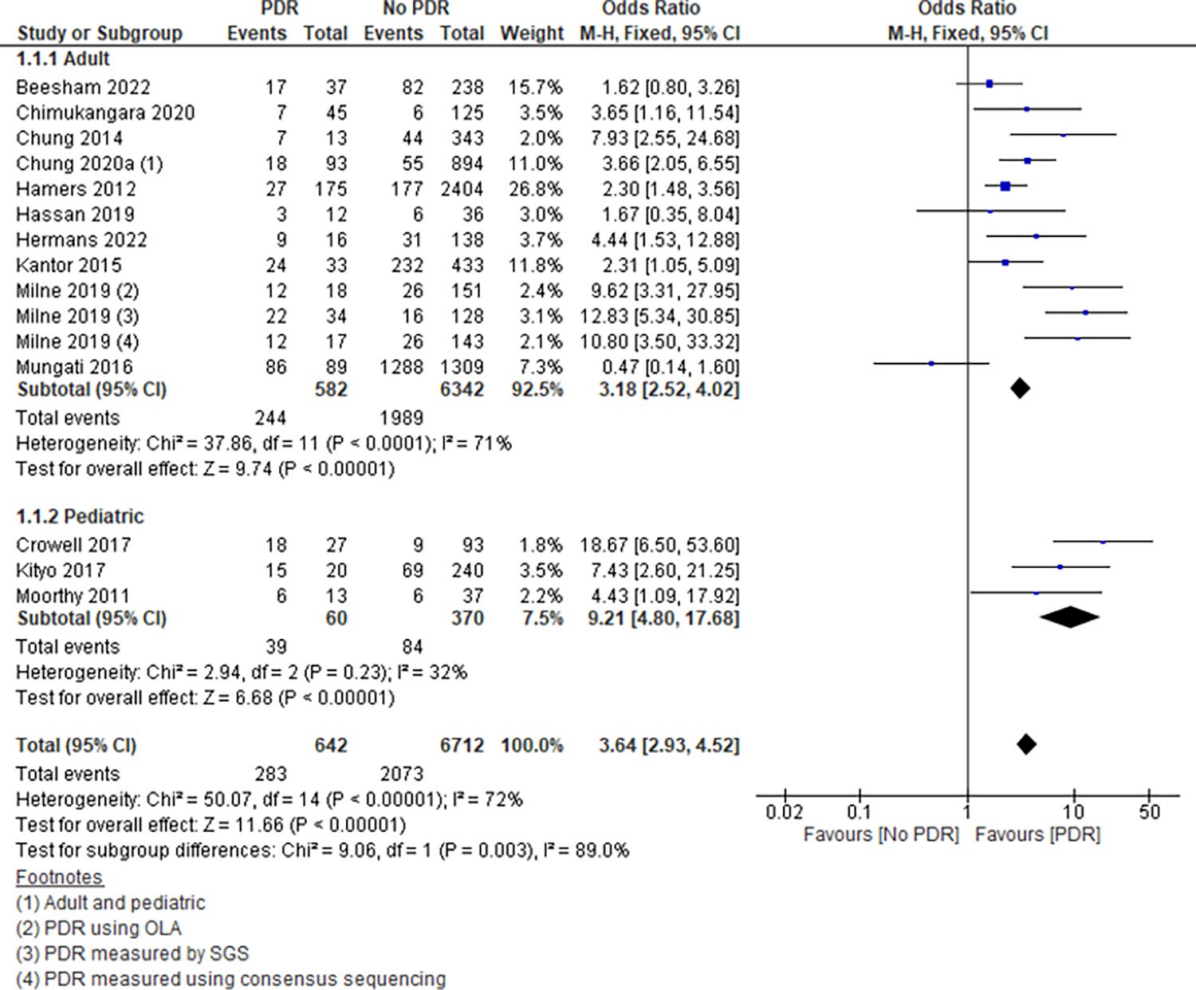
Association between PDR and virological failure (metanalysis).

Overall, the pooled results from the 13 studies showed that those with PDR had higher odds of VF compared to those without PDR, OR 3.64[95% CI 2.93, 4.52]-Fig 3. This indicates that overall, there is evidence that PDR is associated with an increase in virological failure in children and in adults. In adults, the risk was similar to the overall OR 3.18[95% CI 2.52–4.02]. In children, the risk was higher than in adults, with children with PDR having 9 times the odds of VF compared to children without PDR. It should, however, be noted that the number of participants and events in this subgroup of children was considerably less.

#### Association between PDR and VLS

5 studies each showed a reduction in the odds of VLS in those with PDR compared to those without. There was borderline evidence of variability between the individual studies (Heterogeneity test Chi2 11.01, p = 0.05).

**Fig 4:**
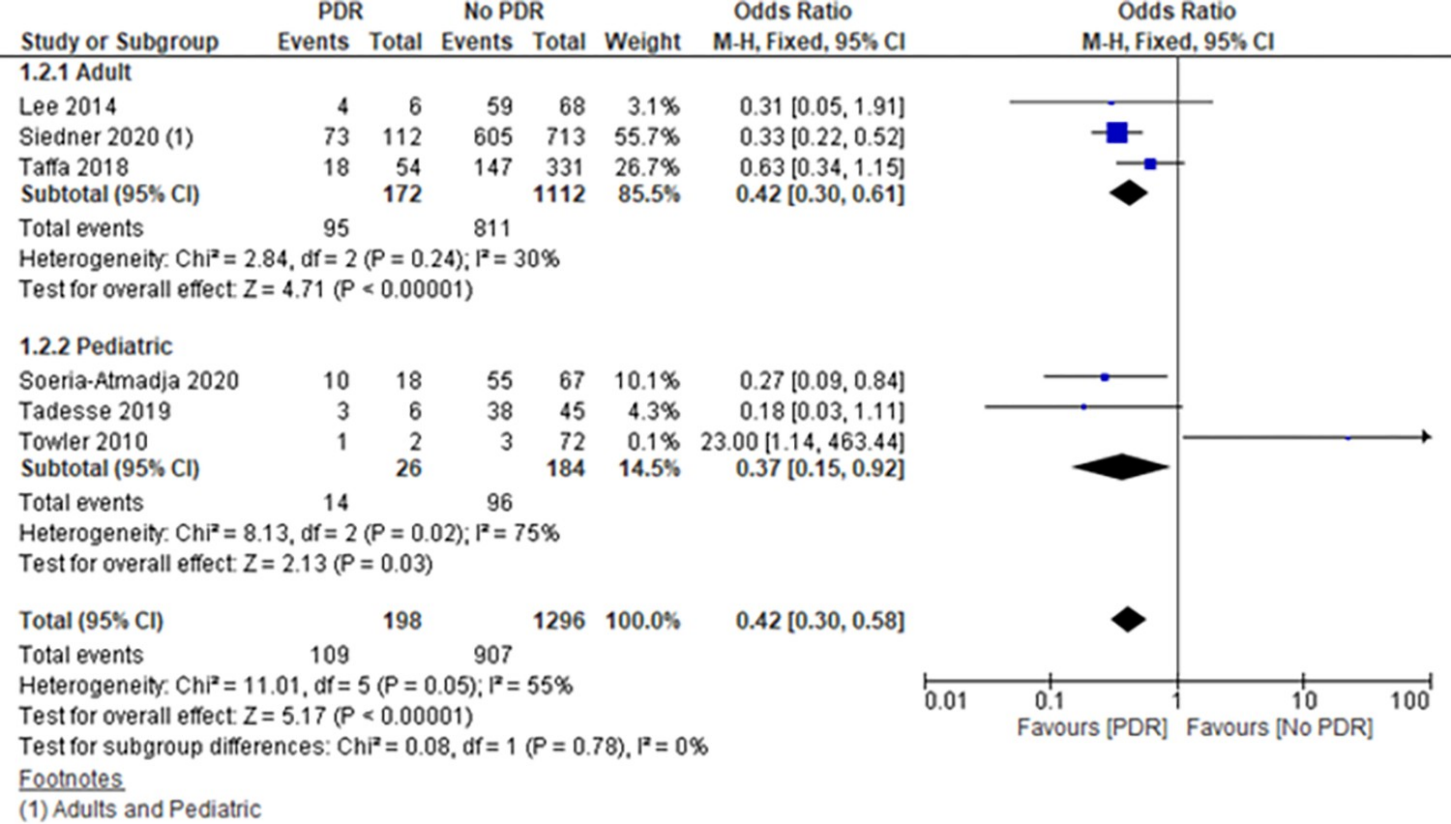
Association between PDR and VLS (metanalysis).

Overall, the pooled results from 6 studies showed a reduction in the odds of VLS in those with PDR compared to those without PDR, OR 0.42 (95% CI 0.30, 0.58) (Fig 4). However, one study showed an increase in VLS for those with PDR compared to those without PDR. It should be noted that this study had few data points, suggesting imprecise estimates.

The odds of VLS in children were similar to those of adults (Fig 4).

#### Reporting biases

It is worth noting that for observational studies, reporting is mostly done for those that have evidence of effect (reporting bias).

#### Certainty of evidence

Most of the studies conducted involved cohort studies which are generally good for looking at associations. The risk of bias from these studies was relatively low since the loss to follow-up was minimal in most of the studies (<20%).

## Discussion

This review indicates that in sub-Saharan Africa, adults and children with PDR have an increased risk of VF and lower “risk” of VLS. This is expected, as the presence of resistant virus in an individual prior to ART initiation could reduce treatment efficacy. It is expected that the PDR, which was present prior to commencement of treatment, would lead to VF for some ARV classes and irrespective of acquired drug resistance, which occurs later. In most of these studies, samples were collected from HIV infected individuals prior to commencement of treatment. The participants were then followed up to evaluate virological outcomes at various time points after starting ARV treatment. The difference in virological failure, between those with PDR and those without PDR could be attributed to the presence of resistant mutations, after adjusting for confounding factors.

These findings were similar to those of another review which showed evidence of association between PDR and virological failure [43]. In the review which included studies in SSA, as well as other regions, the authors showed a higher risk of VF in those with PDR [43]. Bertagnolio et al., also evaluated NNRTI PDR specific effect on virological failure, which was high, and was similar in combinations containing specifically tenofovir. Other reviews showed high levels of PDR in children in SSA but did not look at the association with virological outcomes [44, 45].

The association between PDR and virological failure was more pronounced in children than in adults. This is not unexpected since children are more likely to have transmitted drug resistance from their mothers during pregnancy and birth. In addition, they might have had prior exposure to NNRTI through PMTCT.

In terms of ART class-specific pretreatment drug resistance, majority of the PDR is due to NNRTIs [5–8, 11, 12, 14, 23, 24, 31, 32]. This is not surprising since NVP has been used for a long time in PMTCT programs, which means a considerable number of individuals have had prior exposure to the drug. There were very few published studies that reported on PI specific PDR and we could not find any on INSTI-specific PDR.

It can be argued that the association between PDR and virological suppression observed could be explained by various levels of adherence. However, it is unlikely because after adjusting for adherence, the risk of virological failure associated with PDR was still present in adults [5, 7, 11, 14] and in children [23]. In another study conducted in SA, the rate of suboptimal adherence was quite low and was unlikely to explain the observed association between PDR and VF [31].

Although this review included a number of relevant studies from SSA, some limitations are worth mentioning. First, in some retrieved studies, data on PDR and virological outcomes were not explicitly mentioned. This might affect the representativeness of the studies. The risk of bias was unclear in a number of studies, and this would limit the interpretation of the findings. Other factors which are associated to virological failure other than PDR, for example non-adherence, were usually not available from previous studies. The method of review where the search involved just one database could limit the availability of the studies. There was a lack of studies that presented data disaggregation between men and women to see if the magnitude of the association between PDR and virological failure might be different, since HIV positive women of childbearing age are likely to have had prior exposure to ARVs through the PMTCT program.

Albeit these limitations, there is evidence of an association between PDR and virological failure. A number of implications are worth mentioning in terms of clinical practice and public health. In health facilities in resource limited settings, simple methods can be developed to screen HIV patients prior to treatment initiation in areas where the epidemic has been ongoing for a relatively long time. In this situation, it is likely that a great majority of ART patients might have developed some resistance which might have been transmitted to others (ref). In addition, many HEI might have been exposed to ARVs through PMTCT. In areas with relatively young epidemics, screening for PDR before treatment initiation is unlikely to be of benefit since less patients are expected to have prior exposure to ARVs. In addition, in all settings, all ART patients with high viral load could be systematically screened for PDR as part of their clinical management of high viral load. In terms of public health practice, PDR screening can be included in measurement of impact of ART treatment programs. Surveillance for PDR could be included in HIV drug resistance surveillance. Future research on HIV drug resistance could be geared towards documenting pre-treatment drug resistance in adults and children and documenting PDR.

## Conclusion

In conclusion, this systematic review indicates that PDR increases the risk of virological failure in sub-Saharan Africa. The risk could be reduced by constant monitoring for PDR, mainly NNRTI PDR (PMTCT) and INSTI PDR (transmitted PDR), in addition to ADR in patients who are on ARV. In areas with long lasting HIV epidemics, including PDR screening prior to ART initiation may be of benefit. Surveillance for PDR may be included into HIV drug resistance surveillance.

## Supporting information

S1 Checklist PRISMA 2009 checklist. (DOC)
